# Impact of anxiety and sleep disturbances on postoperative outcomes in male cardiothoracic surgery patients: a multicenter randomized controlled trial evaluating a psychological intervention during the ICU phase

**DOI:** 10.3389/fpsyg.2025.1649765

**Published:** 2025-09-01

**Authors:** Yue Zhang, Dan Li, Xiaofei Bi, Xu Fang, Yang Jing, Baicheng Zhang, Xianglong Kong, Jing Shi

**Affiliations:** ^1^Department of Intensive Care Unit, Second Affiliated Hospital of Harbin Medical University, Harbin, Heilongjiang, China; ^2^Department of Intensive Care Unit, Sixth Affiliated Hospital of Harbin Medical University, Harbin, Heilongjiang, China; ^3^Department of Cardiothoracic Intensive Care Unit, Harbin Medical University Cancer Hospital, Harbin, Heilongjiang, China

**Keywords:** anxiety, cardiothoracic surgery, Cognitive Behavioral Therapy, intensive care, male patients, multicenter study, postoperative recovery, sleep disturbances

## Abstract

**Objective:**

To investigate the prevalence and impact of anxiety and sleep disturbances during the intensive care unit (ICU) stay following cardiothoracic surgery in male patients, and to evaluate the efficacy and feasibility of a structured psychological intervention combining Cognitive Behavioral Therapy for Insomnia (CBT-I) principles with environmental optimization.

**Methods:**

This study was designed as a multicenter, prospective, randomized controlled trial (RCT) conducted from January to April 2025 across three tertiary hospitals. A total of 120 adult male patients who underwent radical surgery for cardiac or lung cancer and were subsequently admitted to the ICU were enrolled. Baseline assessments were performed within 48 h after surgery. Participants were randomly allocated in a 1:1 ratio to either the intervention group (*n* = 60) or the standard care group (*n* = 60) using a computer-generated randomization sequence with concealed allocation. While the standard care group received routine perioperative management, the intervention group additionally received a structured psychological intervention that incorporated components of Cognitive Behavioral Therapy for Insomnia (CBT-I)—including sleep education, relaxation training, and behavioral strategies—along with daily psychological support and environmental optimization measures such as noise reduction, lighting adjustment, and use of sleep-promoting devices.

Primary outcomes included Generalized Anxiety Disorder-7 (GAD-7), Pittsburgh Sleep Quality Index (PSQI), Numeric Rating Scale (NRS) for pain, ICU length of stay, incidence of postoperative complications, and the 30-day postoperative quality of life as measured by the SF-36. Multivariate logistic regression was used to assess the predictive value of anxiety and sleep disturbances on postoperative outcomes.

**Results:**

On postoperative day 3, the intervention group showed significantly lower GAD-7 scores (6.3 ± 1.6 vs. 8.4 ± 2.3, p = 0.016) and PSQI scores (7.5 ± 1.6 vs. 10.2 ± 2.3, *p* < 0.01) compared to the standard care group. Pain scores were also significantly reduced (2.7 ± 1.2 vs. 3.6 ± 1.3, *p* = 0.018). The intervention group had a shorter ICU stay (2.5 ± 0.6 days vs. 3.7 ± 1.2 days, *p* < 0.01), a lower rate of postoperative complications (17% vs. 36%, *p* = 0.033), and significantly better SF-36 scores at 30 days post-surgery (*p* < 0.05). Multivariate logistic regression identified both anxiety and sleep disturbance as independent predictors of postoperative complications (GAD-7: OR = 1.25, 95% CI: 1.03–1.42; PSQI: OR = 1.33, 95% CI: 1.14–1.51).

**Conclusion:**

Anxiety and sleep disturbances are common during the postoperative ICU phase in male patients undergoing cardiothoracic surgery and are significantly associated with pain, complications, and recovery outcomes. Early implementation of a CBT-I–based psychological intervention in the ICU can effectively improve psychological status, shorten ICU stays, and reduce postoperative complications. The intervention is safe and shows high clinical utility, warranting consideration for integration into standardized postoperative care pathways, particularly in high-risk male populations.

**Clinical trial registration:**

The study was retrospectively registered on the Chinese Clinical Trial Registry (ChiCTR) under the identifier ChiCTR240000123.

## Introduction

Cardiothoracic surgery is a highly invasive and physiologically stressful procedure that involves complex perioperative management and a prolonged recovery process. In addition to significant physical trauma, postoperative patients often experience substantial psychological stress. Anxiety and sleep disturbances are among the most common psychological problems in the early postoperative period, particularly during the intensive care unit (ICU) stay, and they have been shown to adversely affect recovery and clinical outcomes (Zergaw et al., 2024; Azimian et al., 2019; Logan and Barksdale, 2008). Studies have demonstrated that postoperative anxiety may activate the hypothalamic-pituitary-adrenal (HPA) axis, leading to elevated cortisol levels, which suppress immune function and impair wound healing. Sleep disturbances can disrupt the deep sleep cycle and inhibit the secretion of growth hormone, further delaying tissue repair and exacerbating the perception of postoperative pain (Irwin, 2019; Addis and Mahalik, 2003; Graham et al., 2023).

In this context, male patients may be especially vulnerable due to gender-specific roles, cultural expectations, and coping mechanisms. Men are more likely to suppress negative emotions and avoid expressing psychological needs, a phenomenon known as “gender silence.” This tendency may contribute to the underdiagnosis of anxiety and sleep disturbances, hinder timely intervention, and increase the risk of postoperative complications, prolonged ICU stays, and delayed overall recovery (Edinger and Means, 2005).

Currently, there is a growing shift from pharmacological approaches to integrated non-pharmacological interventions for addressing postoperative psychological issues. Cognitive Behavioral Therapy for Insomnia (CBT-I) has received increasing attention for its efficacy in postoperative populations. By incorporating sleep education, behavioral restructuring, and relaxation training, CBT-I helps to break the vicious cycle of anxiety and insomnia. Additionally, environmental modifications—such as noise reduction, dim lighting, and the use of earplugs—have been shown to enhance sleep quality and subjective comfort. While previous studies suggest that CBT-I and environmental interventions hold promise, most existing research is limited to single-center studies and general surgical or medical patients. Evidence remains scarce regarding their feasibility and effectiveness in male cardiothoracic patients in the ICU setting, particularly within multicenter frameworks (Kamdar et al., 2012; Redeker et al., 2022).

To address this gap, we conducted a multicenter, prospective study focusing on male patients in the ICU following cardiothoracic surgery. We implemented a comprehensive psychological care pathway that integrated core CBT-I strategies, environmental optimization, and short-term pharmacological support when necessary. This study aimed to systematically evaluate the intervention's impact on postoperative anxiety, sleep disturbances, ICU length of stay, complication rates, and quality of life. Our goal was to provide evidence-based support for early psychological interventions in high-risk postoperative populations and to explore the development of standardized critical care pathways for wider clinical implementation.

## Methods

This multicenter, prospective, randomized controlled trial (RCT) was conducted between January and April 2025 at three tertiary general hospitals in China. The study aimed to evaluate the impact of a structured psychological intervention on anxiety, sleep quality, and postoperative outcomes among male patients admitted to the ICU following cardiothoracic surgery.

Inclusion criteria were as follows:

- Male patients aged ≥18 years;
- Undergoing radical surgery for lung cancer or cardiac disease;
- Admitted to the ICU postoperatively;
- Conscious and cognitively intact;
- Able to complete all study-related assessments.

Exclusion criteria included:

- Severe cognitive impairment;
- History of major psychiatric disorders;
- Multiple-site trauma surgeries.

**Figure 1:**
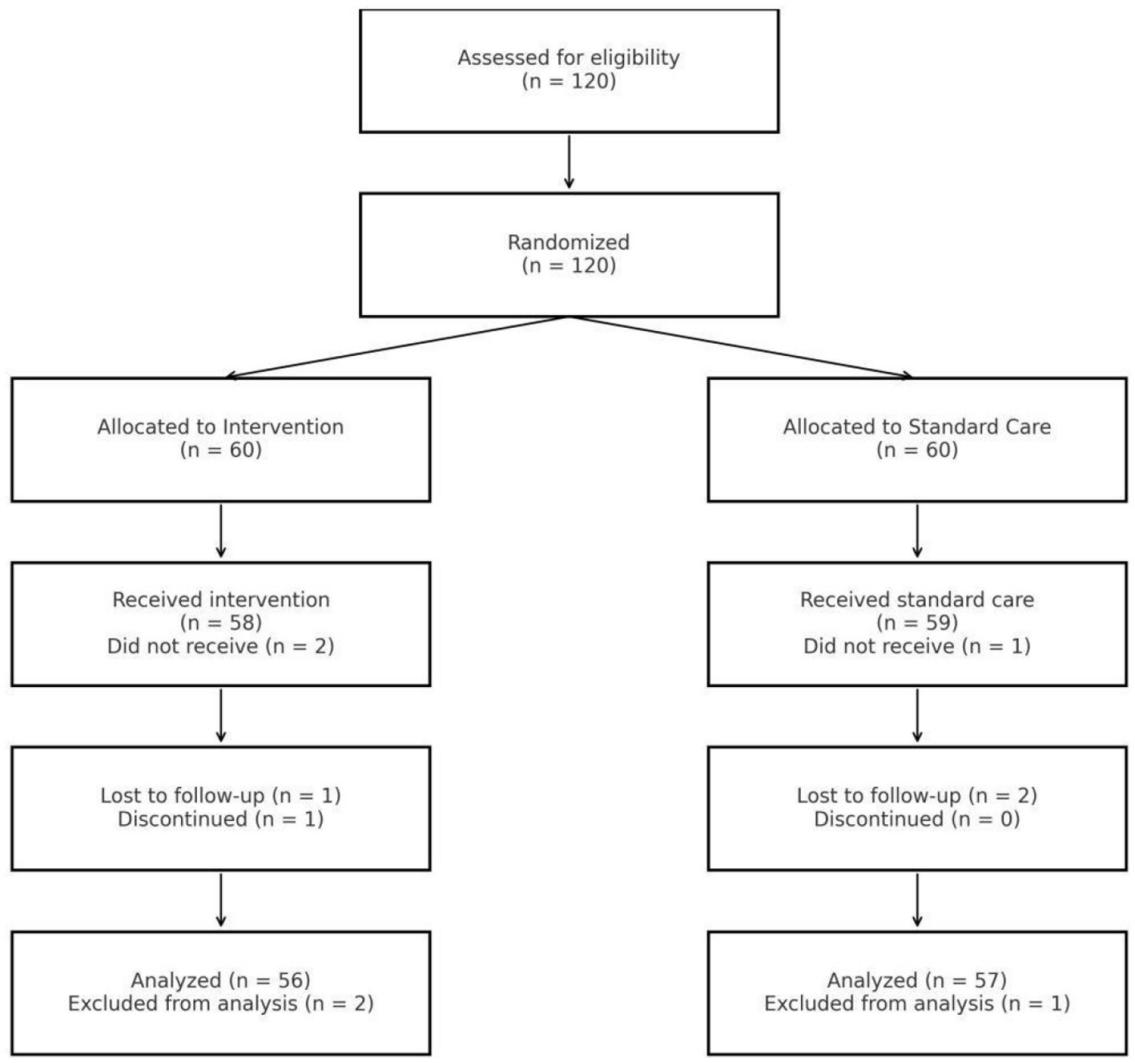
CONSORT flow diagram of participant progress through the study.

A total of 120 eligible participants were enrolled and randomized in a 1:1 ratio to either the intervention group (*n* = 60) or the standard care group (*n* = 60). Randomization was conducted using a computer-generated sequence, and allocation concealment was ensured via sealed, opaque envelopes in accordance with CONSORT guidelines (Figure 1).

Given the nature of the psychological and environmental intervention, blinding of participants was not feasible. However, several measures were taken to reduce potential bias:

- Data collectors and outcome assessors were blinded to group allocation and were not involved in delivering the intervention.
- ICU nurses delivering the intervention received standardized training and followed a unified protocol to ensure consistency across centers.
- Environmental modifications (e.g., earplugs, lighting adjustments) were discreetly implemented to minimize group awareness.
- To reduce contamination, patients were spatially distributed within ICUs, and visual barriers and scheduling were used to minimize interaction between groups.

### Intervention protocol

The psychological intervention combined environmental optimization with non-pharmacological and optional pharmacological strategies, delivered from within 24 h post-surgery and continuing daily throughout the ICU stay.

#### Environmental optimization

- Nighttime noise reduction was achieved by limiting alarms and overhead paging.
- Overhead lights were dimmed from 22:00 to 06:00, with bedside lamps used as needed.
- Disposable soft-foam earplugs were provided nightly.
- All measures were implemented according to a structured nursing protocol and reinforced through training at each center.

#### Cognitive behavioral therapy for insomnia (CBT-I)

- Included brief daily psychological support (20 min/day), sleep education, relaxation training, and behavioral strategies.
- Interventions were administered by trained ICU nurses who completed standardized instruction delivered by a senior clinical psychologist and the principal investigator.
- Protocol fidelity was maintained through weekly audits and centralized supervision across all centers.

#### Pharmacological assistance (if needed)

- For patients with severe sleep disturbances (PSQI >12), short-term use of non-benzodiazepine hypnotics (e.g., eszopiclone, ≤ 3.75 mg/night for ≤ 3 consecutive nights) was permitted.
- Efficacy and adverse events were monitored and recorded.

### Pain management and outcome measures

All patients received standardized analgesia, including intravenous NSAIDs and, when necessary, low-dose opioids. Pain was assessed using the Numerical Rating Scale (NRS) on postoperative days 1, 3, and 7 during rest.

Psychological and clinical outcomes were measured as follows:

- Anxiety and Sleep Quality: assessed using the Generalized Anxiety Disorder-7 (GAD-7) and Pittsburgh Sleep Quality Index (PSQI) at baseline, postoperative day 3 (POD3), and day 7 (POD7).
- Pain: evaluated via the NRS.
- Postoperative complications: defined as new-onset clinical events within 7 days post-surgery requiring medical intervention, including pulmonary infections, arrhythmias, delirium, delayed wound healing, or ICU readmission.
- ICU length of stay: recorded in days.
- Quality of life: assessed at 30 days post-surgery using the 36-Item Short Form Health Survey (SF-36).

All instruments used in the study are validated and widely adopted in clinical research (Spitzer et al., 2006; Buysse et al., 1989; Ware and Sherbourne, 1992):

- GAD-7: high internal consistency (Cronbach's α > 0.85) and test-retest reliability.
- PSQI: adapted for short-term ICU application and validated for sleep quality assessment.
- NRS: reliable and valid for pain evaluation in surgical settings.
- SF-36: a global standard for health-related quality of life assessment.

## Statistical analysis

All statistical analyses were performed using SPSS version 26.0 (IBM Corp., Armonk, NY, USA). Two-tailed tests were applied throughout, and a *p*-value of < 0.05 was considered statistically significant.

Continuous variables (e.g., GAD-7 scores, PSQI scores, ICU length of stay) were expressed as mean ± standard deviation (mean ± SD), and between-group comparisons were conducted using independent sample *t*-tests. Categorical variables (e.g., complication rates, marital status) were presented as counts and percentages [n (%)], and were compared using the chi-square test or Fisher's exact test where appropriate (when the expected frequency was < 5).

To assess the predictive value of anxiety and sleep quality on postoperative outcomes, a multivariable logistic regression model was constructed, with GAD-7 and PSQI scores as independent variables and postoperative complications as the dependent variable. The model was adjusted for potential confounders including age, body mass index (BMI), type of surgery (cardiac vs. thoracic), history of ICU admission, and educational level.

Additionally, a sensitivity analysis was conducted using study center as a stratification variable to evaluate the consistency of the intervention effect across different hospitals and to rule out center-related biases. Prior to analysis, data integrity was verified. For a small proportion of missing data (< 5%), mean imputation was applied; these imputed values were excluded from regression analyses.

We conducted a *post-hoc* power analysis based on the observed difference in GAD-7 scores, which showed sufficient statistical power (1–β = 0.92). Cohen's *d* effect sizes were also calculated to estimate the magnitude of intervention effects, with results indicating large effects for GAD-7 and PSQI, and a medium-to-large effect for NRS.

## Results

A total of 120 male patients meeting the inclusion criteria were enrolled and randomly assigned to the intervention group (*n* = 60) and the standard care group (*n* = 60). There were no significant differences between the two groups in baseline characteristics, including age, body mass index (BMI), type of surgery (cardiac or thoracic), and educational background, indicating good comparability (Table 1).

**Table 1:** Baseline characteristics of patients in the intervention and standard care groups.

**Variable**	**Intervention group (*n* = 60)**	**Standard care group (*n* = 60)**	***P* value**
Mean age (years)	61.3 ± 7.1	61.5 ± 6.8	0.64
Married, *n* (%)	51 (85%)	50 (83%)	0.72
Education ≥ high school, *n* (%)	21 (35%)	24 (40%)	0.67
Employment: employed, *n* (%)	9 (15%)	11 (18%)	0.66
History of smoking, *n* (%)	45 (75%)	48 (80%)	0.52
History of alcohol use, n (%)	26 (43%)	24 (40%)	0.61
BMI (kg/m^2^)	25.8 ± 3.3	26.2 ± 3.6	0.47
History of ICU admission, *n* (%)	4 (6%)	3 (5%)	0.72
History of sleep disorders, *n* (%)	16 (26%)	14 (23%)	0.57
Chronic comorbidities, *n* (%)	31 (52%)	33 (55%)	0.71
Pulmonary resection, *n* (%)	36 (52%)	32 (48%)	0.62
Cardiac surgery, *n* (%)	24 (48%)	28 (52%)	
Duration of anesthesia (hours)	4.8 ± 1.7	4.6 ± 1.4	0.43
Pre-op GAD-7	9.2 ± 2.4	9.4 ± 2.5	0.56
Pre-op PSQI	10.3 ± 2.3	10.9 ± 2.1	0.69

GAD-7, generalized anxiety disorder-7; PSQI, Pittsburgh sleep quality index; BMI, body mass index; ICU, intensive care unit. Values are presented as mean ± standard deviation or *n* (%). Independent *t* tests were used for continuous variables; chi-square tests for categorical variables. *P* < 0.05 was considered statistically significant.

On postoperative day 3, the intervention group demonstrated significantly lower levels of anxiety and improved sleep quality compared to the standard care group. The mean Generalized Anxiety Disorder-7 (GAD-7) score was 6.3 ± 1.6 in the intervention group vs. 8.4 ± 2.3 in the standard care group (*p* = 0.016). The Pittsburgh Sleep Quality Index (PSQI) score was 7.5 ± 1.6 in the intervention group and 10.2 ± 2.3 in the standard care group (*p* < 0.01).

In terms of clinical outcomes, the intervention group reported lower postoperative pain scores on the Numerical Rating Scale (NRS) (2.7 ± 1.2 vs. 3.6 ± 1.3, *p* = 0.018). The mean ICU length of stay was significantly shorter in the intervention group (2.5 ± 0.6 days) compared to the standard care group (3.7 ± 1.2 days, *p* < 0.01). The postoperative complication rate was also significantly lower in the intervention group (17% vs. 36%, *p* = 0.033). Moreover, the SF-36 total score assessed on postoperative day 30 was significantly higher in the intervention group (73.5 ± 8.3 vs. 66.4 ± 9.2, *p* = 0.007), indicating better health-related quality of life (Table 2).

**Table 2:** Comparison of anxiety, sleep quality, pain, ICU stay, postoperative complications, and quality of life between groups.

**Indicator**	**Intervention group (*n* = 60)**	**Standard care group (*n* = 60)**	***P* value**
GAD-7 score (postop day 3)	6.3 ± 1.6	8.4 ± 2.3	0.016
PSQI score (postop day 3)	7.5 ± 1.6	10.2 ± 2.3	< 0.01
NRS pain score (postop day 3)	2.7 ± 1.2	3.6 ± 1.3	0.018
ICU length of stay (days)	2.5 ± 0.6	3.7 ± 1.2	< 0.01
Postoperative complications (%)	17%	36%	0.033
SF-36 score (day 30)	73.5 ± 8.3	66.4 ± 9.2	0.007

NRS, numeric rating scale for pain; SF-36, short form health survey−36. Values are presented as mean ± standard deviation or percentage. *P* values < 0.05 were considered statistically significant.

In the intervention group, 5 patients (8.3%) with a PSQI score >12 on postoperative day 3 were treated with short-term non-benzodiazepine hypnotic medication (eszopiclone, < 3.75 mg/night, for ≤ 3 nights). The average PSQI score in these patients decreased by 3.6 points, and no significant adverse drug reactions were observed. In the standard care group, 8 patients (13.3%) received the same pharmacologic treatment, with an average PSQI reduction of 2.8 points; one patient experienced transient dizziness that resolved without intervention. The between-group difference in sedative use was not statistically significant (*p* = 0.37). None of these patients developed additional postoperative complications, and pharmacologic use did not significantly affect primary outcome measures (Table 3).

**Table 3:** Comparison of short-term hypnotic use and related efficacy between groups.

**Indicator**	**Intervention group (*n* = 60)**	**Standard care group (*n* = 60)**	***P* value**
Use of hypnotics [*n* (%)]	5 (8.3%)	8 (13.3%)	0.37
Medication type	Zopiclone	Zopiclone	—
Duration of use (nights)	≤ 3	≤ 3	—
Dose per administration (mg)	< 3.75	< 3.75	—
Reduction in PSQI (points)	3.6	2.8	—
Adverse events [*n* (%)])	0 (0%)	1 (1.7%)	—
Complications in medicated [*n* (%)])	0 (0%)	0 (0%)	—

Values are presented as number (%) or mean values. “—” indicates no statistical test was applicable.

Multivariate logistic regression analysis was performed to evaluate the predictive effect of anxiety and sleep quality on postoperative complications. Both the GAD-7 score (OR = 1.25, 95% CI: 1.03–1.42, *p* = 0.006) and the PSQI score (OR = 1.33, 95% CI: 1.14–1.51, *p* = 0.002) were identified as independent risk factors for postoperative complications. These associations remained statistically significant after adjusting for age, BMI, type of surgery, education level, and history of ICU admission (Table 4).

**Table 4:** Logistic regression analysis predicting postoperative complications from anxiety and sleep quality.

**Variable**	**OR (95% CI)**	***P* value**
GAD-7 score	1.25 (1.03–1.42)	0.006
PSQI score	1.33 (1.14–1.51)	0.002

OR, odds ratio; CI, confidence interval; GAD-7, generalized anxiety disorder-7; PSQI, Pittsburgh sleep quality index. Higher anxiety and poorer sleep quality were significantly associated with increased risk of complications (*p* < 0.05).

*Post-hoc* power analysis based on the observed between-group difference in GAD-7 scores (mean difference = 2.1, SD ≈ 2.1) revealed an achieved power of 0.92, supporting the adequacy of the sample size for detecting a clinically significant effect. Additionally, effect size estimates further demonstrated the magnitude of the intervention's impact. The between-group effect size for GAD-7 was Cohen's *d* = 0.99, indicating a large effect. For sleep quality assessed by the PSQI, the effect size was *d* = 1.28, representing a very large effect. Pain intensity, measured using the NRS, showed a medium-to-large effect with *d* = 0.73. These results underscore the substantial clinical benefits of the psychological intervention across multiple outcome domains.

## Discussion

This study demonstrates that anxiety and sleep disturbances are prevalent among male patients in the early postoperative period following cardiothoracic surgery in the ICU, and are significantly associated with key recovery outcomes—including ICU length of stay, postoperative complications, and health-related quality of life (HRQoL) (Wang et al., 2020). The implementation of a systematic psychological intervention, particularly one centered on Cognitive Behavioral Therapy for Insomnia (CBT-I), led to substantial improvements in both psychological wellbeing and physical recovery, underscoring the critical role of psychological factors in perioperative management (Hillman et al., 2023).

By postoperative day 3, patients in the intervention group demonstrated significantly lower scores on both the Generalized Anxiety Disorder-7 (GAD-7) and the Pittsburgh Sleep Quality Index (PSQI) compared to the control group (6.3 ± 1.6 vs. 8.4 ± 2.3, *p* = 0.016; 7.5 ± 1.6 vs. 10.2 ± 2.3, *p* < 0.01, respectively). Although both groups' anxiety scores fell within the “mild anxiety” range according to the GAD-7 scale, the statistically significant reduction observed in the intervention group reflects a clinically meaningful improvement. This is especially relevant considering the high-stress environment of the ICU, where even modest decreases in anxiety can have important implications for patient outcomes. The early psychological benefits evident at this relatively short postoperative interval highlight the efficacy of CBT-I-based interventions in mitigating anxiety and improving sleep quality during the critical ICU recovery phase. Given that anxiety is a known risk factor for postoperative complications, these findings underscore the potential of timely psychological interventions to enhance overall recovery and reduce adverse events (Redeker et al., 2022). Moreover, the intervention group experienced reduced pain (NRS: 2.7 ± 1.2 vs. 3.6 ± 1.3, *p* = 0.018), shorter ICU stays (2.5 ± 0.6 days vs. 3.7 ± 1.2 days, *p* < 0.01), and fewer postoperative complications (17% vs. 36%, *p* = 0.033), indicating both subjective and objective benefits of the intervention (Spitzer et al., 2006).

Multivariate logistic regression further confirmed that postoperative anxiety and sleep disturbances independently predicted the occurrence of complications. Each one-point increase in GAD-7 and PSQI scores was associated with a 25% (OR = 1.25, 95% CI: 1.03–1.42, *p* = 0.006) and 33% (OR = 1.33, 95% CI: 1.14–1.51, *p* = 0.002) increase in complication risk, respectively. These findings emphasize that anxiety and sleep issues are not merely subjective symptoms, but critical biopsychosocial factors influencing surgical outcomes and should be addressed systematically (Holzer et al., 2023).

From a mechanistic standpoint, postoperative anxiety may activate the hypothalamic-pituitary-adrenal (HPA) axis, leading to elevated cortisol levels, immunosuppression, delayed wound healing, and a higher risk of infection or other complications (Rabbitts et al., 2020). Similarly, sleep disturbances—especially a lack of deep sleep—can reduce growth hormone secretion, impair tissue repair and immune modulation, and disrupt pain perception and recovery rhythms (Belvederi Murri et al., 2014). The lower complication rate observed in the intervention group (17% vs. 36%) provides clinical validation of these pathophysiological mechanisms.

Importantly, this study also highlights the gender-specific psychological recovery needs of male patients. Compared to women, men are more likely to suppress emotional expression and are less inclined to seek psychological support—a phenomenon often described as “gendered silence.” This may result in underdiagnosed and undertreated anxiety and insomnia in male surgical patients (van Vught et al., 2009). By introducing structured daily psychological support in the intervention group, this study created an emotional outlet and provided practical sleep strategies, contributing to improved recovery in this at-risk population.

Improvements in sleep and reduced anxiety during early postoperative recovery are known to enhance physical functioning, emotional wellbeing, and social engagement—core dimensions of HRQoL measured by the SF-36. Literature suggests that early psychological stabilization positively influences adaptation to illness and long-term health perceptions (Savard et al., 2005). These mechanisms may partly explain the improved HRQoL scores observed in the intervention group.

CBT-I, a globally endorsed non-pharmacological intervention, incorporates core elements such as sleep education, relaxation training, and behavioral modification to interrupt the vicious cycle of anxiety, insomnia, and poor recovery. The addition of environmental optimization measures (e.g., noise reduction, dim lighting, use of earplugs) further mitigated the overstimulating ICU environment, enhancing sleep quality and psychological stability (Mackenzie et al., 2006; Savard et al., 2005).

The study also included a short-term pharmacological component for patients with severe insomnia (PSQI >12), using non-benzodiazepine hypnotics (eszopiclone < 3.75 mg/night, ≤ 3 nights). Among the five patients in the intervention group who received medication, sleep quality improved significantly without observed adverse effects or increased complication risk (Manber et al., 2008). In contrast, eight patients in the standard care group also received medication, but their improvement was less pronounced, and some continued to experience moderate anxiety. This suggests that pharmacological treatment alone is insufficient without concurrent psychological support (Devlin et al., 2018; Liang et al., 2019). These findings support a stepped-care intervention model—anchored in psychological therapy with targeted pharmacologic bridging when necessary. Although no serious side effects or dependency were observed, the lack of long-term follow-up precludes definitive conclusions on sustained medication use, highlighting the importance of cautious, time-limited application.

While this study benefits from a multicenter, prospective design with standardized intervention protocols and strong translational potential, several limitations must be acknowledged. First, the sample size was relatively modest, potentially limiting the power of subgroup analyses. Second, the follow-up duration was restricted to 30 days postoperatively, which is insufficient to assess long-term HRQoL, psychological trajectory, or readmission risk. Third, some core outcomes relied on self-reported scales, which may be influenced by patient cooperation or emotional state.

One notable limitation of this study is the use of the Pittsburgh Sleep Quality Index (PSQI) to assess sleep quality in an ICU setting. While the PSQI is traditionally designed to evaluate sleep patterns over a 1-month interval, we adapted the tool to reflect the preceding 3-day period, following methods reported in previous ICU-based research. Despite this adaptation, the use of a subjective and originally long-term assessment tool may introduce measurement bias or limit sensitivity to rapid changes in sleep quality during the acute postoperative phase. Future studies should consider incorporating objective sleep assessment methods, such as actigraphy or polysomnography, to enhance the accuracy and clinical relevance of sleep measurements in critically ill patients. Additionally, future studies will include female and non-binary patients to investigate potential gender differences in psychological recovery. We further propose exploring digital delivery models of CBT-I, artificial intelligence–based screening tools, and integrating psychological interventions into enhanced recovery after surgery (ERAS) protocols. These strategies may enhance the scalability and routine implementation of mental health support as a standard component of perioperative care.

## Conclusion

Anxiety and sleep disturbances are critical psychological factors affecting postoperative recovery in male patients undergoing cardiothoracic surgery. This study demonstrates that a comprehensive psychological care intervention—centered on Cognitive Behavioral Therapy for Insomnia (CBT-I)—administered during the ICU period can effectively alleviate anxiety, improve sleep quality, and significantly enhance postoperative outcomes. This non-pharmacological approach is both safe and feasible, with strong applicability across multiple centers. It is therefore recommended that CBT-I–based psychological care be integrated into standardized critical care protocols as an essential component of postoperative recovery management.
